# The Effect of Sleep Quality on Pain in Chilean Individuals with Musculoskeletal Disorders

**DOI:** 10.3390/ijerph182111370

**Published:** 2021-10-29

**Authors:** Claudio Bascour-Sandoval, Hellen Belmar-Arriagada, Javier Albayay, Claudia Lacoste-Abarzua, Diego Bielefeldt-Astudillo, Rubén Gajardo-Burgos, Mirko Vidal-Torres, Germán Gálvez-García

**Affiliations:** 1 Grupo de Investigación Clínica en Kinesiología, Carrera de Kinesiología, Universidad Autónoma de Chile, Temuco 4780000, Chile; claudio.bascour@ufrontera.cl (C.B.-S.); claudia.lacoste@uautonoma.cl (C.L.-A.); diego.bielefeldt@uautonoma.cl (D.B.-A.); 2 Departamento de Medicina Interna, Universidad de La Frontera, Temuco 4780000, Chile; 3 Programa de Magíster en Terapia Física con menciones, Universidad de La Frontera, Temuco 4780000, Chile; hellen.belmar.a@gmail.com; 4 Facultad de Salud y Ciencias Sociales, Universidad de las Américas, Concepción 4030000, Chile; 5 Center for Mind/Brain Sciences, University of Trento, 38068 Rovereto, Italy; javier.albayay@unitn.it; 6 Instituto de Aparato Locomotor y Rehabilitación, Universidad Austral de Chile, Valdivia 5090000, Chile; ruben.gajardo@docentes.uach.cl (R.G.-B.); mirko.vidal@uach.cl (M.V.-T.); 7 Centro Universitario de Rehabilitación, Universidad Austral de Chile, Valdivia 5090000, Chile; 8 Departamento de Psicología, Universidad de La Frontera, Temuco 4780000, Chile; 9 Département de Psychologie Cognitive & Neuropsychologie, Institut de Psychologie, Laboratoire d’Étude des Mécanismes Cognitifs, Université Lyon b, 69676 Lyon, France

**Keywords:** sleep quality, musculoskeletal disorders, musculoskeletal pain, chronic pain, pain

## Abstract

Poor sleep quality (SQ) negatively affects pain associated with musculoskeletal disorders (MSD). As the level of economic development of a country determines its sanitary conditions, these can influence the sleep–pain relationship; therefore, it is relevant to generate evidence in the population with MSD in developing countries. This cross-sectional study sought to determine the effect of poor SQ on pain in Chilean individuals with MSD, controlling for sex and duration of pain (in months). Method: A total of 228 individuals were included. SQ was measured with the Pittsburg Sleep Quality Index (PSQI), pain (intensity, interference and distress relative to pain) was measured with visual analog scales. Structural equation modeling (SEM) was performed to analyze the effect of SQ on pain. Results: A high frequency of poor SQ was present in the studied group, and was more prevalent in women. The SEM model evidenced that poor SQ predicts greater pain. Sex influences sleep quality and pain, but not pain duration. Conclusions: These findings indicate that poor SQ predicts higher pain in MSD and that women exhibit worse SQ and more significant pain than men. Our findings support that SQ should be considered in the comprehensive approach to pain in individuals with MSD.

## 1. Introduction

Musculoskeletal disorders (MSD) are a major public health issue [1] characterized by the presence of pain and frequently associated with sleep disturbances resulting in poor sleep quality (SQ) [2]. Thus, there is increasing research interest in the link between SQ and pain in individuals affected by MSD.

Although some studies have described the relationship between SQ and pain as bidirectional [3], recent evidence indicates that SQ predicts pain more strongly than pain predicts SQ [4,5,6]. Poor SQ can alter key processes in pain perception. For instance, total and partial sleep deprivation has been shown to interfere with pain processing, inducing hyperalgesia in pain-free subjects and individuals with MSD [5,7]. Furthermore, poor SQ can contribute to acute pain continuing, acting as a risk factor for developing chronic pain [8,9].

Other relevant factors, such as sex and pain duration, influence SQ and pain perception in individuals affected by MSD. Women report a higher frequency of poor SQ and pain than men [10,11], and a longer duration of pain has been associated with poor SQ [10] and higher perception of pain [12]. However, other studies have not established this connection and it remains controversial [13,14].

Most of the literature on the relationship between SQ and pain in individuals with MSD has been generated in high-income, English-speaking countries, such as the United States, Canada, the United Kingdom, Finland, Israel, and Norway, with a few studies in non-native English-speaking countries like Brazil [4,15,16]. Mainly, investigations in Hispano-American countries concerning the relationship between SQ and pain in individuals with MSD are limited and inconclusive [17,18,19]. For instance, two studies found that poor SQ is associated with more musculoskeletal pain in individuals with chronic MSD [17] and sleep disorders [18]. However, the study by Covarrubias et al. [17] lacks clarity about the treatment of missing data and reporting of their results is deficient. Mariños et al. [18], for their part, present a poor characterization of the sample and non-validated instruments for their variables. The study by Navarro-Aquino et al. [19] found no significant association between SQ and pain but, similar to the others mentioned, presents an incomplete statistical analysis and poor methodological and results reporting. In addition, the three only measured one dimension of pain (i.e., intensity), which together with methodological flaws, can significantly limit their scope.

Due to the preceding works, the investigation by Stubbs et al. [20] becomes relevant. These authors carried out a study based on the general population in different countries (without a specific diagnosis), reporting variation in the SQ–pain association between middle-income and low-income countries. They highlight the need to continue generating studies in various socioeconomic and cultural realities given that differences between countries (i.e., access to analgesics or sleep medications, or non-pharmacological interventions such as physiotherapy/psychological treatment) and cultures (i.e., traditions or religious beliefs) may modify this relationship [20]. This is highly relevant when assessing the SQ–pain association in individuals with MSD.

Due to the negative impact that MSD represent, it is essential to identify factors that affect pain and represent a barrier in their treatment, especially those that are modifiable, such as a poor SQ [21,22]. This would guide therapeutic strategies, especially in individuals with chronic pain, where pharmacological treatment has achieved modest results, increasing the need to improve non-pharmacological approaches [22,23,24] and comprehensive management [24,25].

Here, we endeavor to determine the effect of SQ on pain in Chilean individuals affected by MSD, accounting for the effect of sex and pain duration. We approached the measurement of pain as multifactorial, considering intensity, distress and interference in daily activities [26]. We expected to find poor SQ to be associated with higher levels of pain and being female, and pain of longer duration to be associated with poor SQ and higher levels of pain.

## 2. Materials and Methods

### 2.1. Participants

A cross-sectional and analytical design was used. Individuals with either acute or chronic MSD were recruited by non-probabilistic consecutive sampling. Namely, each consecutive eligible patient who presented for MSD care during a defined period of time (from July 2019 to April 2020) was eligible to take part in this study [27,28]. They underwent physical therapy in two university rehabilitation centers specialized in MSD in southern Chile. The major MSD (i.e., low back pain, neck pain, osteoarthritis) and other MSD (e.g., disorders of synovium and tendon and other soft tissue disorders) are most frequently addressed. Upon entering rehabilitation (i.e., physical therapy), individuals were invited to participate provided that they met the following inclusion criteria: aged 18 years or over, speak Spanish and present a medical diagnosis of an MSD. Individuals who presented a neurological and/or cognitive condition that did not allow an adequate evaluation to be carried out were excluded. Likewise, individuals with severe uncompensated visual or hearing impairment were excluded. A total of 272 admissions were recorded during that period (87 admissions were in the city of Valdivia and 195 in the city of Temuco), of which 228 agreed to participate (mean age = 49.02 years old, SD = 17.93; mean education = 13.2 years of education, SD = 3.21; 72.8% were women) and made up the final sample of this study. The participants were asked to provide sociodemographic information concerning age, sex, years of education, work status, marital status, personal monthly income, and MSD type. The descriptive characteristics of the sample are presented according to sex in Table 1.

### 2.2. Instruments

#### 2.2.1. Pain

Pain was measured multidimensionally using a visual analog scale (VAS), since it is a highly reproducible instrument, quick and easy to apply [26]. The scale consisted of a horizontal 100 mm line, whose ends were labeled as the extremes of different expressions of pain.

Intensity of pain: The left end of the VAS indicated absence of pain (i.e., “no pain”) and the right the greatest intensity (i.e., “worst pain imaginable”). The individual was asked to mark the point that best indicated perceived pain intensity to measure its distance with a ruler later. The intensity of the pain was measured at rest (i.e., without movement), movement (i.e., most painful movement), and the average pain of the last 7 days.

Interference related to pain: This describes how the pain interferes with the individual’s daily activities [26]. The left end of the VAS indicated the absence of interference (i.e., “without interference”), and the right indicated the greatest interference (i.e., “unable to carry out their activities”).

Distress relative to pain: This is described as a multifactorial unpleasant emotional experience of a psychological nature (cognitive, behavioral, and emotional), social or even spiritual, due to persistent or recurrent pain [26]. Participants were asked to rate pain-related distress experienced in the past week on the VAS. The left end indicated the absence of distress (i.e., “without distress relative to pain”), and the right indicated the most significant distress (i.e., “maximum distress relative to pain”).

Duration of pain: This was evaluated by asking, “How many months have you suffered the current pain?”

#### 2.2.2. Sleep Quality

SQ was measured using the Spanish version of the Pittsburgh Sleep Quality Index (PSQI) [29], a commonly used tool, practical and brief, which measures SQ in the last month. The PSQI consists of 24 self-rated questions, of which 19 are included to obtain the overall SQ score, which is expressed on a scale of 0–21 points; a higher score indicates a worse SQ. From the global score, the evaluated individuals can be classified as having “Good SQ” (≤5 PSQI) or “Poor SQ” (>5 PSQI), with a sensitivity of 89.6% and a specificity of 86.5%, according to Buysse et al. [30]. The Spanish version has demonstrated good internal consistency (α = 0.78) [29].

### 2.3. Procedure

During admission to rehabilitation (i.e., physical therapy), the participants were informed about the purpose of this study and their participation. All participants endorsed their decision to participate in the study by signing the informed consent. The professional in charge of applying the instruments was specially instructed before starting the investigation (i.e., using clear and understandable language) and to reduce the possible biases associated with self-report instruments, adequate time and space were set aside in the interviews. The participants were asked to complete the self-reported questionnaires on a tablet. The data provided were collected and managed using the REDcap^®^ electronic recording tool hosted by the Universidad de La Frontera. In some instances, the participant did not feel comfortable handling the tablet; in such cases, the interviewer used it. All tests concerning the present study were carried out during the first physical therapy session, namely, before the patients started any treatment. The patients then followed the treatments that the physical therapist at the centers deemed appropriate regardless of the study.

The anonymity of the participant was protected. Each has a secret ID known only by the researchers, which was used throughout the process, including the possible publications that could derive from this investigation. This study followed the Guidelines of the Declaration of Helsinki [31] and was approved by the Ethics Committee of the Universidad Autónoma de Chile (No. 62-18).

### 2.4. Data Analysis

Descriptive statistics were used. The Shapiro–Wilk test showed that the quantitative variables did not comply with the assumption of normality. Thus, medians and interquartile range (IQR) were estimated. Quantitative and qualitative variables were compared according to sex by means of Mann–Whitney U test and chi-squared test (χ^2^), respectively.

**Figure 1:**
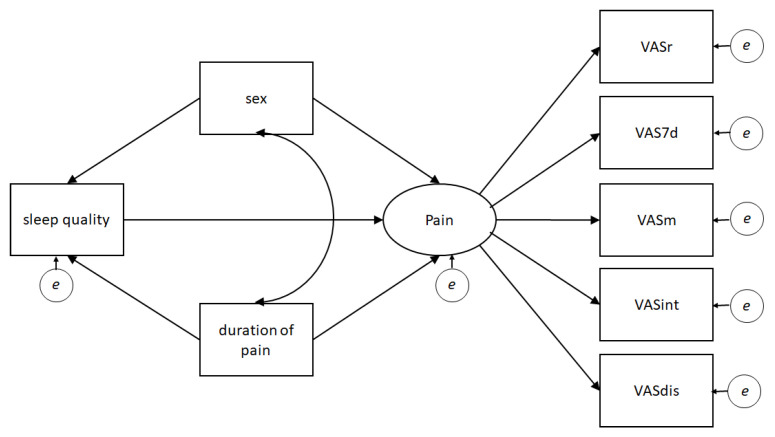
Hypothesized structural equation model. Note: The oval represents the latent variable (i.e., pain), the rectangles the indicators (i.e., measured variables), and the small circles the errors. VAS, visual analog scale; VASr, VAS at rest; VAS7d, average VAS 7 days; VASm, VAS at movement; VASint, VAS pain interference; VASdis, VAS distress relative to pain.

Structural equation modeling (SEM) was performed to analyze the SQ effect on pain, controlling for sex and duration of pain. SEM comprises a set of robust multivariate analysis techniques—with greater statistical power as compared to other statistical techniques—that allow to test causal relationships among variables while also correcting for measurement error [32]. In brief, SEM allowed us to model simultaneously the relationships between the variables of interest and their directionality based on the proposed hypothesis. Accordingly, the latent variable pain was created. In line with Treede et al. [26], the variable Pain included the following factors: pain intensity (static, at movement and average of the last 7 days), interference of pain in daily activities and distress relative to pain. A model was analyzed in which the SQ variable predicts the pain variable based on the current evidence describing that SQ predicts pain more strongly than pain predicts SQ [4,5,6]. Additionally, the variables sex and duration of pain were included in the model to control SQ and pain. The hypothesized SEM is described graphically in Figure 1. For the sample size calculation, 10 participants were estimated for every free parameter; thus, a minimum of 180 individuals were considered for the present study [33].

Correlational analyses were used to evaluate the association between SQ (i.e., PSQI score) and pain (each variable included in the SEM model) among individuals with MSD by means of Spearman’s Rho.

Mardia’s test was performed to analyze multivariate normality. As the assumption of normality was not fulfilled (*p* < 0.001), a maximum likelihood estimation with Santorra–Bentler correction was performed. The goodness-of-fit index was determined considering χ^2^, and as limit values the comparative fit index (CFI ≥ 0.95), Tucker–Lewis index (TLI ≥ 0.90), the root mean square error of approximation (RMSEA ≤ 0.06), and the standardized root mean square residual (SRMR ≤ 0.08), according to the cut-off scores established by Hu and Bentler [34]. There were no missing data in the study.

The statistical analysis was performed with the Stata 14 software.

## 3. Results

Descriptive results by sex are presented in Table 2. Older age and more significant pain (all variables/measures) were observed in women, except pain duration, which showed no differences between men and women. In addition, women showed higher PSQI scores (i.e., poor SQ) and higher frequencies of poor SQ. Table 3 presents Spearman’s Rho correlation coefficient.

**Figure 2:**
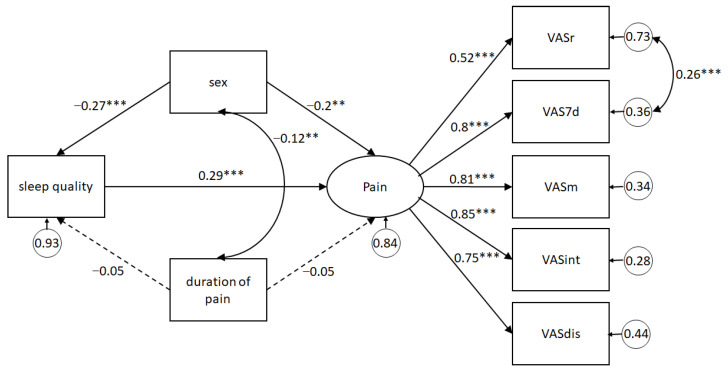
Structural equation model predicting pain from sleep quality, controlling for sex and pain duration. Note: Statistics are standardized regression coefficients. Dotted lines represent non-significant relations; bold lines represent significant paths. The curve lines correspond to covariation between variables or their errors. VASr, VAS at rest; VAS7d, average VAS 7 days; VASm, VAS at movement; VASint, VAS pain interference; VASdis, VAS distress relative to pain. * *p* < 0.05, ** *p* < 0.01, *** *p* < 0.001.

As a first step, the latent variable pain was evaluated according to its goodness-of-fit indicators. A covariance between static pain intensity and mean pain intensity of the last 7 days was modeled. The model provided a good fit for the data χ^2^(4) = 3.88, *p* = 0.423; CFI = 1.0; TLI = 1.0; RMSEA < 0.001, 90% CI (0.00, 0.10); SRMR = 0.01. Next, the entire model was evaluated, showing its results with standardized coefficients in Figure 2, indicating that poor SQ predicts greater pain (β = 0.29, 95% CI (0.16, 0.42), *p* < 0.001). Sex had a direct effect on SQ and pain. Given that the variable sex was coded as 0 for women and 1 for men, it is evidenced that women have worse SQ than men (β = −0.27, 95% CI (−0.39, −0.15), *p* < 0.001) and greater pain (β = −0.20, 95% CI (−0.34, −0.05), *p* < 0.001). Pain duration has no effect on SQ (β = −0.05, 95% CI (−0.19, 0.08), *p* = 0.44) nor on pain (β = 0.05, 95% CI (−0.05, 0.16), *p* = 0.33). Even so, pain duration covaried with sex (β = −0.12, 95% CI (−0.20, 0.04), *p* = 0.005). The fit of this model was excellent (χ^2^(16) = 17.68, *p* = 0.34; CFI = 0.99; TLI = 0.99; RMSEA = 0.02, 90% IC (0.00, 0.07); SRMR = 0.02). Hence, it may be suggested that SQ has an effect on pain, that women show poor SQ and greater pain than men, and lastly, that pain duration has no effect on SQ or pain in these Chilean adults with MSD.

## 4. Discussion

The present study aimed to determine the SQ effect on pain in Chilean individuals with MSD, controlling for sex and pain duration. Our findings support the hypothesis that SQ predicts greater pain in individuals with MSD, which are in line with previous studies [12,35]. The Hispanic-American studies, conducted in Mexico [17,19] and Peru [18], specifically, provide descriptive information about the prevalence of SQ and pain in the studied population. Furthermore, they present poor methodological and results reporting in terms of writing and incomplete information [17,18], and one of them had a small sample size, which could lead to not finding a relation between SQ and pain [19]. Furthermore, Mariños et al. [18] measured specific sleep impairments (apnea, insomnia, and somnolence) without accurate instruments. Thus, our investigation contributes to the corpus of knowledge on this relation, specifically to understanding of the link between SQ and pain in individuals with MSD in a developing country like Chile, expanding the radius of evidence beyond developed and English-speaking countries as proposed by Stubbs et al. [20] and Afolalu et al. [4]. This is relevant given that, unlike developed countries, in Chile there is marked inequity in access to health, especially mental health (where sleep impairments are framed) [36] as well as in the access and quality of treatment for MSD, which the latter are perceived by our population as deficient or modest [36,37].

A high frequency of poor SQ has been described in the population with MSD, reporting up to 86% [38], comparable with the frequency found in our sample (82.89%). Such data support the idea that SQ should be considered in the clinical management of individuals with MSD.

The variable sex had a direct effect on SQ and pain. Thus, women presented a higher percentage of poor SQ than men (86.75% vs. 72.58%, respectively). This is in line with the results of various previous studies that describe women as having higher levels of poor SQ in objective and subjective measurements [5,10]. This can be explained by the alteration of sleep architecture caused by menstrual cycles, pregnancy and menopause, in addition to higher risk factors for poor SQ such as depression and anxiety [39]. Regarding the effect of sex on pain, our results show a higher level of pain in women in agreement with previous studies [10,40,41]. This difference could be due to multiple mechanisms that include differences in endogenous opioid function, sexual hormone effects, affective/cognitive influences [42], as well as the contribution of social factors such as gender role stereotypes [43,44] and other responsibilities related to particular stages of life, such as those related to childcare or work–life balance [45].

Pain duration had no effect on SQ or on pain. It should be noted that the evidence of the effect of pain duration on SQ is still controversial. On the one hand, studies by Nicassio et al. [14] and Luyster et al. [13] in individuals with rheumatoid arthritis (RA), a disease characterized by the presence of pain, found no relation between the disease duration and PSQI score (i.e., SQ). On the other hand, Sezgin et al. [10] showed that pain duration had adverse effects on SQ for individuals with chronic low back pain (CLBP). Similarly, evidence on the effect of pain duration on pain intensity is contradictory. The data from Nicassio et al. [14], Luyster et al. [13] and Van Looveren et al. [6] showed no effect, while O’Brien et al. [12] described that duration of pain predicts a more significant pain in CLBP individuals. The difference between our results and those of Sezgin et al. [10] and O’Brien et al. [12] regarding the effect of pain duration on SQ and pain could be down to the heterogeneity of diagnoses and pain durations of the individuals included in our study (35.96% acute vs. 64.04% chronic). In addition, it should be considered that individuals with CLBP and RA are classified differently according to the mechanism underlying the pain. CLBP is classified as primary chronic pain, which is not associated with tissue damage but with complex multidimensional pain characteristics [26,46]. Pain due to RA is classified as secondary chronic pain and exhibits characteristics of inflammatory pain, relating to a basal pathology. This difference in underlying mechanisms of pain could modify the SQ–pain association. Likewise, it should be noted that Nicassio et al. [14] and Luyster et al. [13] considered the disease duration and not precisely the duration of pain. However, more research is needed to clarify this aspect.

Further, our results revealed a direct and moderate relationship between SQ and both pain intensity and pain interference, although lower than those reported in previous studies (i.e., between 0.37 and 0.55) [13,14,23,38]. This could be due to the studies mentioned above focusing particularly on the association between SQ and chronic pain. It is possible that the inclusion of individuals with acute pain in this study (35.96%) might have decreased the magnitude of association between SQ and pain, suggesting that the relationship is stronger between SQ and chronic pain.

One aspect to highlight in the present study is the SEM analysis used. This robust statistic technique allows for model latent variables, reporting the common variation of several indicators contingent on a larger construct. In this vein, following Treede et al. [26], this study addressed the measurement of pain from a multidimensional perspective and considered intensity, distress and interference in daily activities to focus on characteristics of severity, temporality and psychosocial factors. This difference with other studies that use only one dimension of pain (often intensity) is fundamental. This is related to the International Association of the Study of Pain (IASP) proposal, which calls on healthcare professionals to approach pain from a more complex perspective [26,47].

Our study is not free of limitations. First, we used non-probability sampling, which might affect the generalizability of our findings. Indeed, the relationship between SQ and pain may vary in other populations affected by acute or chronic pain (i.e., fibromyalgia, headache/migraine, idiopathic pain disorders, and cancer-related pain). In addition, it should be considered that sleep habits of specific populations (i.e., people who work night shifts, such as healthcare professionals) could modulate the effect of poor SQ on pain. This aspect should be clarified in future studies. Second, our sample was composed mostly of women, although a resent systematic review revealed that a significant number of studies include mainly women in their samples [16]. Like many other studies, our sample includes a relatively large number of students. Thus, the generalization of the present results must be contrasted against more heterogeneous samples. However, it should be noted that the current findings might be particularly relevant for the vast corpus of studies that include a large number of students in their samples [48]. Furthermore, the fact that a large number of students were recruited was not determined by the consecutive sampling used in the present study but by the nature of the rehabilitation centers (i.e., university rehabilitation centers specialized in MSD), whose regular patients include a significant proportion of students with distinctive characteristics such as insufficient sleep [49] and high degree of stress. Finally, we only included subjective, self-reported sleep measures. Although this might be considered a limitation, some authors indicate that self-reported measures of SQ should be considered the gold standard over polysomnography (PSG) based on the idea that PSG can present some variability and imprecisions. It only has the capacity to capture a particular moment of a condition that often spans a lifetime [12,50]. Finally, it should be emphasized that pain is a complex experience that includes sensory, emotional, cognitive, and social factors [51,52,53] that should be addressed in future research on the association of sleep and pain for a richer and more holistic interpretation.

## 5. Conclusions

All in all, our findings suggest that poor SQ predicts higher pain levels, highlighting an important factor to be considered in the approach of pain in individuals with MSD and, possibly, chronic pain prevention. These findings are relevant for those who suffer from MSD and for the healthcare professionals who treat them. We consider these results a starting point in understanding the association between SQ and pain in developing countries. Future research could address this relationship in clinical populations (e.g., shoulder pain, osteoarthritis, tendinopathy), implement greater control of confounding factors, and determine whether the improvement of poor SQ has a positive impact on pain in individuals with MSD.

## Figures and Tables

**Table 1:** Sociodemographic characteristics by sex.

Variable	Women *n* = 166	Men *n* = 62	Total *n* = 228
Age, Md (IQR)	53.5 (40–66)	42 (25–58)	51.5 (30–64)
Years of education, Md (IQR)	12 (12–15)	12 (12–16)	12 (12–15)
Work Status, *n* (%)			
Student	24 (14.5)	17 (27.4)	41 (18.0)
Housewife	69 (41.6)	0 (0.0)	69 (30.3)
Retired	9 (5.4)	7 (11.3)	16 (7.0)
Healthcare Services	9 (5.4)	2 (3.2)	11 (4.8)
Service Occupation	22 (13.3)	9 (14.5)	31 (13.6)
Office and Administration	17 (10.2)	15 (24.2)	32 (14.0)
Transport	1 (0.6)	3 (4.8)	4 (1.8)
Education and Library	11 (6.6)	3 (4.8)	14 (6.1)
Others	4 (2.4)	6 (9.7)	10 (4.4)
Marital Status, *n* (%)			
Single	65 (39.2)	32 (51.6)	97 (42.5)
Married	52 (31.3)	22 (35.5)	74 (32.5)
Divorced	19 (11.4)	3 (4.8)	22 (9.7)
Separated	17 (10.2)	0 (0.0)	17 (7.5)
Widow/Widower	8 (4.8)	0 (0.0)	8 (3.5)
Co-habitation	5 (3.0)	5 (8.1)	10 (4.4)
Monthly Income in USD, *n* (%)			
Less than 250	30 (18.1)	7 (11.3)	37 (16.2)
251–500	76 (45.8)	20 (32.3)	96 (42.1)
501–1000	34 (20.5)	14 (22.6)	48 (21.1)
1001–1500	12 (7.2)	10 (16.1)	22 (9.7)
More than 1500	14 (8.4)	11 (17.7)	25 (11.0)
Type of MSD, *n* (%)			
Cervical	10 (6.0)	3 (4.8)	13 (5.7)
Dorsal-Lumbar	28 (16.9)	8 (12.9)	36 (15.8)
UL No-Trauma	49 (29.5)	15 (24.2)	64 (28.1)
UL Trauma	6 (3.6)	3 (4.8)	9 (3.9)
LL No-Trauma	66 (39.8)	25 (40.3)	91 (39.9)
LL Trauma	7 (4.2)	8 (12.9)	15 (6.6)

Note: Md, median; IQR, interquartile range (upper and lower limit); UL, upper limb; LL, lower limb.

**Table 2:** Clinical characteristics by sex.

Variable	Women *n* = 166	Men *n* = 62	*p-*Value	Total *n* = 228
Pain intensity at rest, Md (IQR)	29.5 (5–54)	15.5 (0–32)	0.003 **	25 (3–49)
Pain intensity at movement, Md (IQR)	73 (53–86)	64.5 (33–84)	0.048 *	71 (50–85)
Pain intensity 7 days, Md (IQR)	62.5 (41–76)	46.5 (19–70)	0.001 **	58 (32.5–75)
Interference of daily activities, Md (IQR)	65 (49–84)	50 (18–72)	<0.001 ***	61 (42–80)
Distress relative to pain, Md (IQR)	74 (52–88)	45 (20–81)	<0.001 ***	71.5 (41–86.5)
Duration of pain (months), Md (IQR)	6 (2–24)	6 (3–12)	0.377	6 (2–13.5)
Total score PSQI, Md (IQR)	10 (7–12)	7 (5–9)	<0.001 ***	9 (6–11)
PSQI categories, *n* (%)				
Good sleep quality	22 (13.25)	17 (27.42)	0.011 *	39 (17.11)
Poor sleep quality	144 (86.75)	45 (72.58)		189 (82.89)

Note: Md, median; IQR, interquartile range (upper and lower limit); PSQI, Pittsburgh Sleep Quality Index; Mann–Whitney U test for continuous variables and χ2 for categorical variables. * *p* < 0.05, ** *p* < 0.01, *** *p* < 0.001.

**Table 3:** Bivariate correlations among the variables included in the structural equation modeling.

Variable	1	2	3	4	5	6	7	8
1. PSQI								
2. Sex	0.27 ***							
3. Duration	0.002	−0.06						
4. VASe	0.22 ***	−0.20 **	−0.004					
5. VAS7d	0.26 ***	−0.22 **	0.01	0.54 ***				
6. VASdyn	0.25 ***	−0.13 *	0.02	0.42 ***	0.66 ***			
7. VASint	0.31 ***	−0.23 ***	0.04	0.39 ***	0.61 ***	0.63 ***		
8. VASdis	0.23 ***	−0.22 ***	0.01	0.33 ***	0.53 ***	0.57 ***	0.65 ***	

Note: PSQI, Pittsburgh Sleep Quality Index; Duration, pain durations; VASe, VAS at rest; VAS7d, average VAS 7 days; VASdyn, VAS at movement; VASint, VAS pain interference; VASdis, VAS distress relative to pain. Code for women = 0 and men = 1. * *p* < 0.05, ** *p* < 0.01, *** *p* < 0.001.

## Data Availability

The data that support the findings of this study are available from the corresponding author, upon reasonable request.
